# Relationship Between Hematologic Inflammatory Markers and Glycemic Control in Type 2 Diabetes Mellitus Patients in a Tertiary Hospital in Nigeria

**DOI:** 10.7759/cureus.68186

**Published:** 2024-08-30

**Authors:** Lemchukwu Amaeshi, Olufunto O Kalejaiye, Oluwarotimi B Olopade, Michael Kehinde

**Affiliations:** 1 Clinical Hematology, Internal Medicine, Lagos University Teaching Hospital, Lagos, NGA; 2 Clinical Hematology, Internal Medicine, College of Medicine, University of Lagos and Lagos University Teaching Hospital, Lagos, NGA; 3 Endocrinology, Diabetes and Metabolism, Internal Medicine, Lagos University Teaching Hospital, Lagos, NGA

**Keywords:** subclinical inflammation, type 2 diabetes mellitus, mean platelet volume, platelet-lymphocyte ratio, neutrophil-lymphocyte ratio, glycated hemoglobin

## Abstract

Introduction: Hematologic inflammatory biomarkers derived from a full blood count (FBC) are elevated in Type 2 diabetes mellitus (DM). In low- and middle-income countries like Nigeria, a FBC is an affordable and easily available test, even in rural areas. Glycated hemoglobin (HbA1c), a measure of glycemic control, has been found to correlate with hematologic inflammatory markers. In Nigeria, where health care is expensive and patients essentially pay out of pocket, a more affordable and accessible alternative to HbA1c in determining glycemic control is needed. Therefore, this study aimed to determine the relationship between Hb A1c and hematologic inflammatory biomarkers, neutrophil-lymphocyte ratio (NLR), platelet-lymphocyte ratio (PLR), and mean platelet volume (MPV), in predicting glycemic control.

Methods: This was a six-month study of 109 patients with Type 2 DM in a tertiary hospital in Lagos. The patients' HbA1c and FBC were measured. NLR, PLR, and MPV were derived from the FBC values. We categorized the patients based on glycemic control. Spearman correlation analysis was used to determine the relationship between HbA1c and the inflammatory biomarkers.

Results: There was no significant difference in NLR, PLR, and MPV between optimal and suboptimal controlled diabetic patients. Spearman’s correlation analysis showed no significant association between NLR, PLR, MPV, and HbA1c in the patients (NLR: r=0.027, P=0.680; PLR: r=-0.091, P=0.356; MPV: r=-0.032, P=0.744).

Conclusion: The inflammatory markers studied had no significant relationship with HbA1c and might not help monitor glycemic control in Type 2 DM patients.

## Introduction

Diabetes mellitus (DM) is a metabolic disease caused by abnormalities in insulin secretion and function, leading to chronic hyperglycemia [1]. The 2021 International Diabetes Federation statistics estimated that 537 million people have diabetes, a number that is projected to soar to about 783 million by 2045 if no interventions are made [2]. The majority of these patients are in low- and middle-income countries, where socioeconomic and sociopolitical factors have led to limited access to health care and, ultimately, late disease presentation. By 2045, 55 million people will be living with diabetes in Africa, a staggering 150% increase from 22 million in 2021 [2]. Nigeria, in particular, is witnessing a rapid rise in the prevalence of diabetes. In 1963, a study reported the disease prevalence as <1%, a figure that has now escalated to 5.5% in 2018 [3,4]. With the country’s rapid population growth, increasing life expectancy, and Type 2 DM risk factors such as obesity and a sedentary lifestyle, the prevalence of this disease is set to continue its alarming ascent. As medical professionals, researchers, and policymakers, your role in addressing this epidemic is crucial.

Type 2 DM is the most common type of DM globally. In Nigeria, it accounts for 90% of all cases of DM [5]. The pathogenesis of Type 2 DM is characterized by insulin resistance, which develops due to subclinical inflammation [6,7]. Studies have found increased inflammatory markers such as C-reactive protein (CRP), interleukin-6 (IL-6), and soluble tumor necrosis factor-alpha (TNF-alpha) in Type 2 DM patients [6,8]. Recently, there has been a growing interest in the role of inflammatory hematologic parameters such as neutrophils, lymphocytes, and platelets in predicting and prognosticating clinical outcomes of non-communicable diseases such as cardiovascular diseases, cancer, and diabetes [9,10]. Observational studies have looked at the predictive and prognostic role of full blood count (FBC) derivatives such as neutrophil-lymphocyte ratio (NLR), platelet-lymphocyte ratio (PLR), and mean platelet volume (MPV) in several non-communicable diseases. These have been termed inflammatory biomarkers and are comparable to traditional inflammatory markers such as C-reactive protein as markers of subclinical inflammation [11,12]. Several studies have found elevated levels of these markers in patients with Type 2 DM compared to non-diabetic individuals.

Glycated hemoglobin (HbA1c) is a marker of glycemic control in diabetic patients and a determinant of microvascular complications. Some studies have found a correlation between HbA1c and these hematologic inflammatory biomarkers [13,14]. However, in Nigeria, where the burden of Type 2 DM is rising, there is a dearth of studies on the relationship between these markers and HbA1c. In a country where 70% of the citizens live below the poverty line [15], these hematologic inflammatory biomarkers, which are easily derived from FBC parameters, may be an available, affordable, and accessible alternative to HbA1c in monitoring glycemic control and possibly predicting the development of diabetic complications [16]. Our study aimed to determine the relationship between these markers and HbA1c in assessing glycemic control in adult patients with Type 2 DM.

"This article was previously posted to the Research Square preprint server on July 12, 2023."

## Materials and methods

Study design

This cross-sectional study was conducted between January 14, 2019 and June 13, 2019, at the Endocrinology Outpatient Clinic of Lagos University Teaching Hospital, Lagos, Nigeria. 

Study population

The participants of this study were known patients with Type 2 DM, aged 18 years and above. We used convenience sampling to select the patients. Pregnant patients, patients with Type 1 DM, those with an ongoing infection or acute inflammation (CRP >10 mg/L) [17], those with a co-existing hematologic disorder, and those who had recent medical interventions (in the previous three months) were excluded. Written informed consent was obtained from each patient before inclusion into the study. We divided the patients into two groups based on their HbA1c levels. Group A (optimal control) were those with HbA1c <7%, and group B (suboptimal control) had HbA1c >7% [17,18]. An interviewer-administered questionnaire was used to obtain information on sociodemographic characteristics, duration of Type 2 DM, other co-morbidities, and medication history.

Sample size calculation

The sample size was determined using the formula for comparison of means [19]:

$n=\frac{{(u+v)}^2(\sigma_1^2+\sigma_0^2)}{\left(\mu_1-\mu_{0\ }\right)^2}$

Where *n*=sample size per group; *u*=one-sided percentage point of the normal distribution corresponding to 100%, the power; Power=90%, *u*=1.28; *v*=percentage point of the normal distribution corresponding to the (two-sided) significance level; significance level=5%, *v*=1.96; µ_1_=mean PLR in those with diabetes=122.45 [20]; µ_0_=mean PLR in those without diabetes=100.55 [20]; σ_1_=standard deviation (SD) of PLR in those with diabetes=37.43 [20]; σ_0_=SD of PLR in those without diabetes=48.14 [20].

The approximate minimum sample size was 81. To increase the study's power, we enrolled 134 patients with diabetes.

Laboratory procedures

An automated hematology analyzer determined the full blood count and the mean platelet volume. NLR and PLR were calculated by dividing the total absolute neutrophil count by the total lymphocyte count and the total platelet count by the total lymphocyte count, respectively. We assayed the C-reactive protein and HbA1c levels using the Finecare™ Quantitative Kit and FIA8000/FIA8600 Quantitative Immunoassay Analyzer. 

Statistical analysis

Data were analyzed using IBM SPSS Statistics for Windows, Version 21.0 (Released 2012; IBM Corp., Armonk, New York, United States). Data distributions were tested for normality using the Shapiro-Wilk normality tests. Continuous variables were expressed as mean±SD for normally distributed data and median and interquartile range for skewed data. Categorical variables were represented as frequency and percentages. Continuous variables of hematological inflammatory markers were assessed for their association with glycemic control (two-level categorical variable of optimal vs. suboptimal) using a Student's t-test. A P-value of <0.05 was considered statistically significant. 

Ethical approval

The study was approved by the Lagos University Teaching Hospital, Health Research Ethics Committee (approval number: NHREC: 19/12/2008a).

## Results

One hundred thirty-four patients with Type 2 DM were enrolled, but only 109 were analyzed. Twenty-five (18.6%) were excluded from the analysis because their CRP levels were >10 mg/L, and peripheral blood film showed platelet clumps.

Sociodemographic and clinical characteristics of study participants

Table 1 represents the sociodemographic and clinical characteristics of the patients. The mean age of the patients was 58±11 years. All patients were on antidiabetic medications, out of which 96 (88.1%) were on metformin, 47 (43.1%) on sulfonylureas, and 38 (34.8%) on insulin. Of the 109 patients, 73 (66.9%) were on antihypertensive medications, 57 (52.2%) on statins, and 36 (33.0%) on antiplatelets.

**Table 1:** Sociodemographic and clinical characteristics of patients. SD: standard deviation; n: frequency; BMI: body mass index; GLP-1: glucagon-like peptide-1; DPP4: dipeptidyl peptidase 4.

Variables	Patients (*N*=109)
Age, mean±SD	58±11 years
Sex	
Males, *n* (%)	41 (37.6)
Females, *n* (%)	68 (62.3)
Mean duration of diabetes (years)	9.3±9
Hypertension, *n* (%)	73 (66.9)
Mean BMI, kg/m^2^	29.7±6.6
Medications	*n* (%)
Metformin	96 (88.1)
Sulfonylureas	47 (43.1)
Insulin	38 (34.8)
Thiazolidinediones	3 (2.7)
DPP4 inhibitors	18 (16.5)
GLP-1 agonists	1 (0.9)
Antihypertensives	73 (66.9)
Statins	57 (52.2)
Antiplatelets	36 (33.0)

Relationship between hematologic and biochemical parameters and glycemic control (HbA1c) in study participants with Type 2 DM

**Figure 1:**
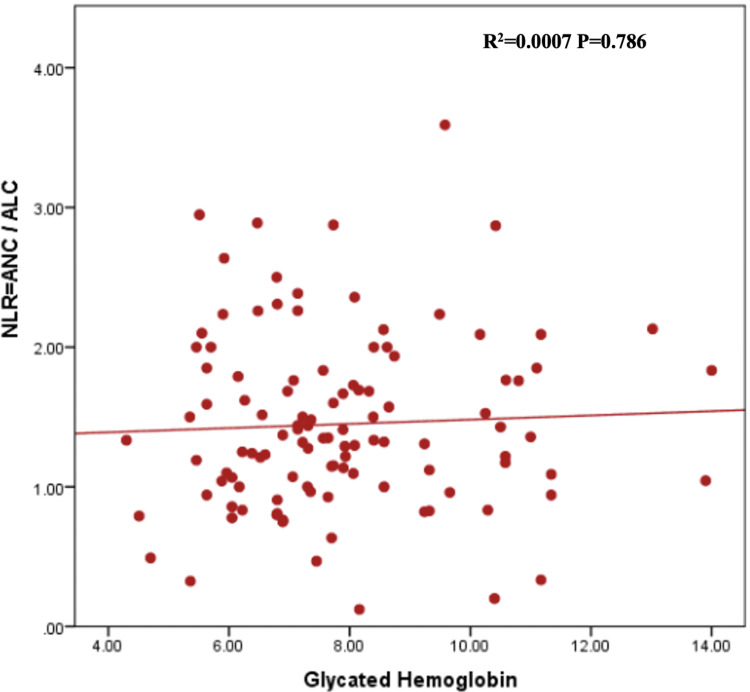
Scatter plot graph representing the correlation between NLR and HbA1c of the patients. NLR: neutrophil-lymphocyte ratio; ANC: absolute neutrophil count; ALC: absolute lymphocyte count; HbA1c: glycated hemoglobin.

**Figure 2:**
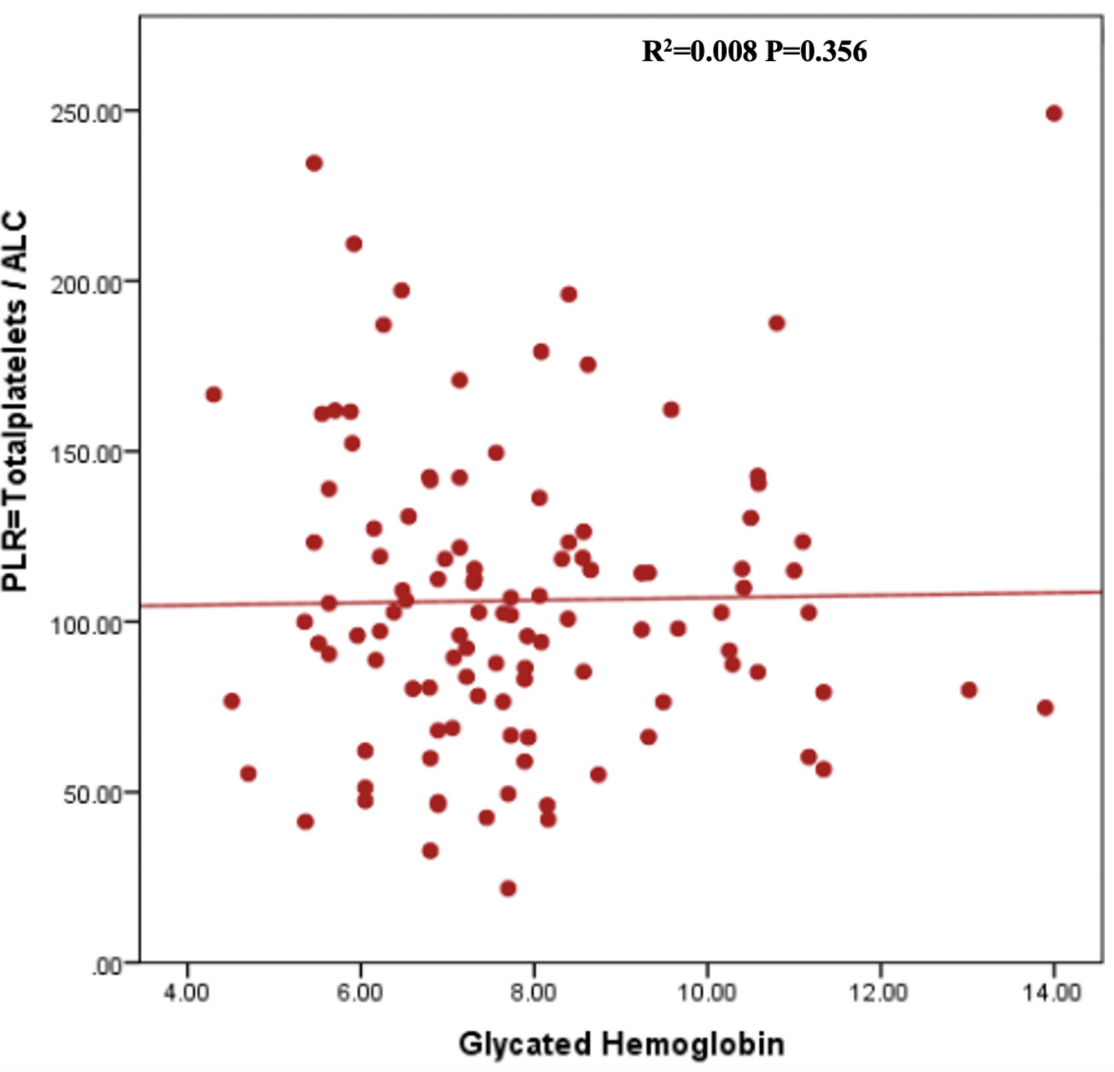
Scatter plot graph representing the correlation between PLR and HbA1c of the patients. ALC: absolute lymphocyte count; PLR: platelet-lymphocyte ratio; HbA1c: glycated hemoglobin.

**Figure 3:**
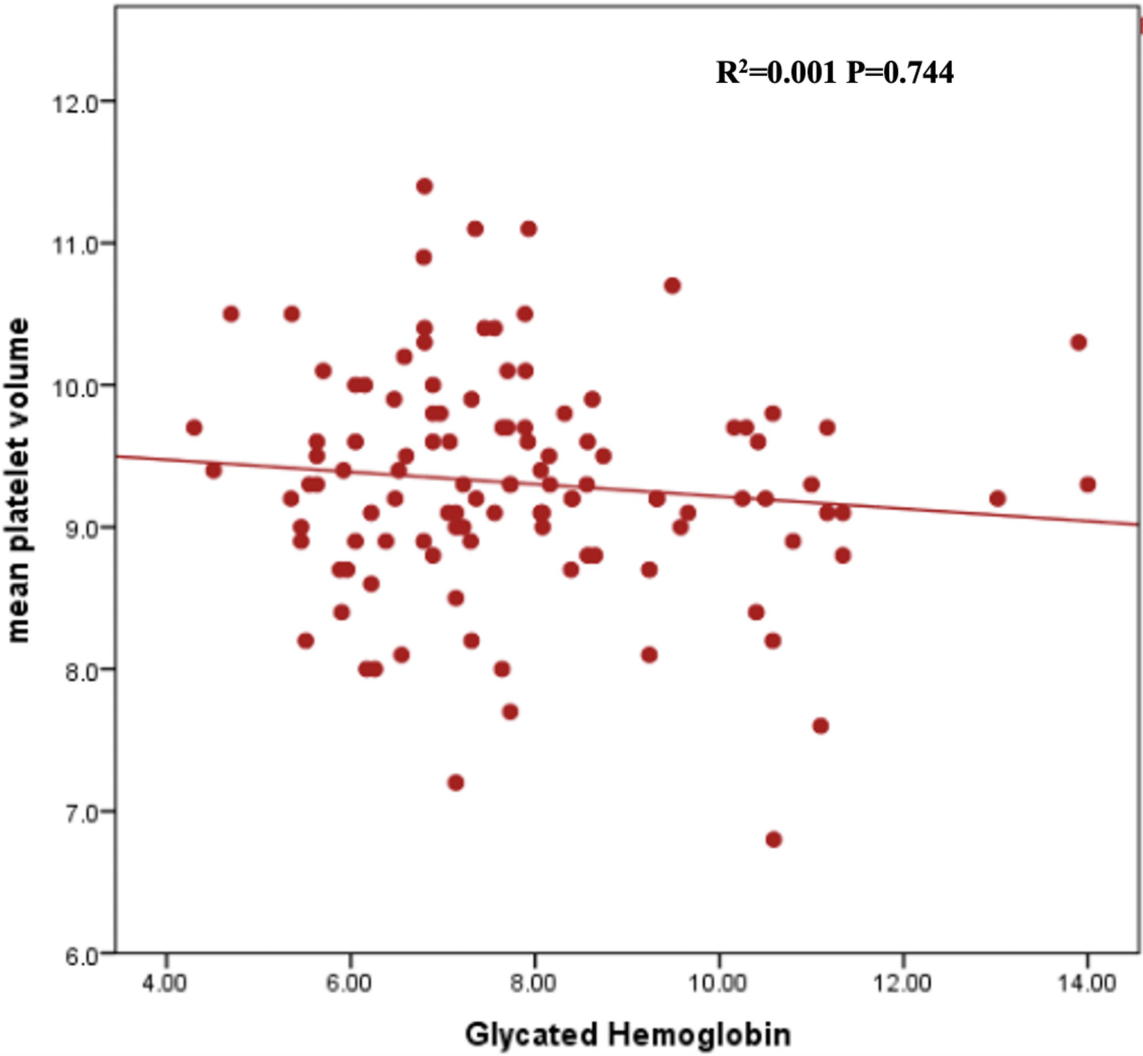
Scatter plot graph representing the correlation between MPV and HbA1c of the patients. MPV: mean platelet volume; HbA1c: glycated hemoglobin.

In Table 2, the mean total white cell count was higher among diabetes patients with suboptimal glycemic control, i.e., HbA1c >7%, compared with optimal control, i.e., HbA1c <7%; however, the difference was not statistically significant [(6.3+1.89) vs. (5.9+1.84) x 10^9^ cells/L, P=0.305]. The mean NLR was approximately equal in both groups of Type 2DM patients [(1.48+0.60) vs. (1.47+0.65) x 10^9^ cells/L, P=0.950]. Although not statistically significant, the mean PLR was higher among those with optimal glycemic control compared to those with suboptimal glycemic control [109.3 (88.8-152.4) vs. 102.5 (80.0-121.8) x 10^9^ cells/L, P= 0.393]. Spearman’s rank correlation coefficient visualized the correlation between HbA1c and NLR, PLR, and MPV, which showed no correlation of NLR, PLR, and MPV with Hb A1c (Figures 1-3).

**Table 2:** Comparison of hematologic and biochemical parameters based on glycemic control among patients (N=109). *n*: frequency; WBC: white blood cell; ALC: absolute lymphocyte count; ANC: absolute neutrophil count; PLT: platelet count; PLR: platelet-lymphocyte ratio; MPV: mean platelet volume; HbA1c: glycated hemoglobin; IQR: interquartile range; fL: fentoliter; IQR: interquartile range. Apart from total PLT and PLR, which are represented as median (IQR), the other variables are represented as mean±SD. Test statistic: Student's t-test compared the means of the two groups. A P-value <0.05 was considered statistically significant.

Parameters	Suboptimal glycemic control (HbA1c >7.0%) (*n*=69)	Optimal glycemic control (HbA1c <7.0%) (*n*=40)	Test statistic	P-value
Total WBC count x 10^9^/L	6.3±1.89	5.9±1.84	1.030	0.305
ALC x 10^9^/L	2.4±0.88	2.34±0.92	0.400	0.690
ANC x 10^9^/L	3.3±1.28	3.1±1.20	0.828	0.409
Total PLT count x 10^9^/L	225 (190-264)	232 (180-286)	-0.177	0.860
NLR	1.48±0.60	1.47±0.65	0.063	0.950
PLR, median (IQR)	102.5 (80.0-121.8)	109.3 (88.8-152.4)	-1.134	0.257
MPV (fL)	9.3±0.8	9.4±0.8	-0.714	0.477

## Discussion

We sought to determine the relationship between hematologic inflammatory biomarkers and HbA1c in patients with Type 2 DM. The goal was to use these markers to monitor glycemic control in communities of low socioeconomic status, where HbA1c testing may not be available and affordable. Our study did not find a significant correlation between these markers and HbA1c.

Similar studies that assessed the relationship between NLR and HbA1c did find a correlation. A study by Hussain et al. on using NLR as an assessment tool for glycemic control found a positive correlation between NLR and HbA1c in diabetic patients [12]. A study by Sefil et al. on the relationship between NLR and blood glucose regulation in diabetic patients in Turkey also found a positive association between NLR and HbA1c [13]. Compared to our study, these studies excluded patients on antiplatelet and lipid-lowering medications. Studies have reported the anti-inflammatory properties of antiplatelet agents such as aspirin and glucose-lowering agents like metformin apart from their primary pharmacologic actions [21,22]. Aspirin reduces inflammation by lowering CRP, byproduct of interleukin-6 (IL-6), an inflammatory mediator [23]. Metformin, a first-line glucose-lowering agent used by over 90% of the patients in our study, reduces inflammation by inhibiting the signaling of NF-κB, a pro-inflammatory transcription factor in vascular tissues and hepatocytes, and the expression of IL-6 and IL-1β, thereby ultimately reducing NLR [24,25]. The anti-inflammatory effects of the medications used might have been responsible for our study's poor association between NLR and HbA1c. This may also explain why there was no significant difference in the white cell count parameters between patients with optimal and suboptimal glycemic control. We included patients on these medications to assess the usefulness of these markers in predicting glycemic control in day-to-day clinical practice. Most Type 2 diabetic patients in Nigeria and other African countries are on statins and antiplatelets, in addition to metformin and other glucose-lowering agents [26].

We also did not find a correlation between PLR and HbA1c. Subclinical inflammation expressed as elevated interleukin-1, IL-6, and TNF-alpha levels stimulates increased production of thrombopoietin, leading to increased platelet production [27]. Elevated PLR has been reported in patients with diabetes [9,20]. The use of aspirin could explain the lack of correlation between these parameters. Few studies have investigated the relationship between glycemic control and PLR. Atak et al. studied PLR as a novel inflammatory marker in 63 patients with Type 2 DM and found a positive correlation with HbA1c [28]. They excluded patients on aspirin, which is known to have anti-inflammatory and antiplatelet properties and may lower PLR.

Similar to our findings with NLR and PLR, we found no correlation between MPV and HbA1c. A few studies also found no relationship between MPV and HbA1c [29-31]. In contrast, a retrospective study by Lippi et al., involving over 4,000 patients, examined the relationship between MPV and HbA1c and reported a positive correlation between HbA1c and MPV [32]. Ozder and Eker, in Turkey, studied MPV in patients with Type 2 DM and impaired fasting plasma glucose and found a positive correlation between MPV and HbA1c. They excluded patients on antiplatelets in their study [33]. Besides the larger sample size of their studies, the exclusion of patients on routine medications for diabetes, including glucose-lowering medications, aspirin, and statins, may explain their findings. MPV represents the average size of platelets. Platelets with high MPV are larger, younger, and more reactive and occur in chronic inflammatory states [14,34]. In diabetes, hyperglycemia creates a chronic inflammatory milieu, which increases oxidative stress and can lead to endothelial dysfunction [35,36]. These factors increase platelet reactivity, leading to thrombopoiesis and higher MPV in diabetic patients [29,37]. A retrospective study by Sivri et al. investigated the effects of statins on MPV and found that statins lower MPV irrespective of lipid levels [38]. Statins reduce MPV by inhibiting the action of CD36, a glycoprotein that promotes platelet activation and thrombosis when bound to oxidized low-density lipoprotein [39]. A prospective study by Dolasık et al. among newly diagnosed patients with Type 2 DM examined the effect of metformin on MPV and found a significant reduction in MPV from the initiation of metformin to six months following treatment, but still no correlation with HbA1c [40]. Metformin reduces leukocyte oxidative stress on the vascular endothelium, reducing platelet activation, platelet hyperreactivity, and consequently MPV [41].

Our study had limitations. Because it was cross-sectional, the data we collected represent only a specific point in time. As a result, we cannot draw conclusions about the long-term effects of glycemic control on these markers. Additionally, the small sample size may have made it difficult to detect significant differences in the subgroups we analyzed. A larger, multicenter, prospective study involving a more extensive population of Type 2 diabetes patients, including those who are newly diagnosed and have not yet received treatment, would be valuable for understanding the impact of high blood sugar on these biomarkers.

## Conclusions

There was no relationship between hematologic inflammatory markers and glycated hemoglobin in patients with Type 2 DM, and therefore, they may not be helpful in directly assessing glycemic control in patients with diabetes in day-to-day clinical practice. The routine medications that individuals with diabetes take have anti-inflammatory properties, which lower the levels of these biomarkers irrespective of glycemic control.

## References

[1] American Diabetes Association (2010). Diagnosis and classification of diabetes mellitus. Diabetes Care.

[2] Nigeria diabetes report 2000 — 2045 [Internet]. https://diabetesatlas.org/data/en/country/145/ng.html.

[3] Kinnear TW (1963). The pattern of diabetes mellitus in a Nigerian teaching hospital. East Afr Med J.

[4] Uloko AE, Musa BM, Ramalan MA (2018). Prevalence and risk factors for diabetes mellitus in Nigeria: a systematic review and meta-analysis. Diabetes Ther.

[5] Ogbera AO, Ekpebegh C (2014). Diabetes mellitus in Nigeria: the past, present and future. World J Diabetes.

[6] Temelkova-Kurktschiev T, Siegert G, Bergmann S, Henkel E, Koehler C, Jaross W, Hanefeld M (2002). Subclinical inflammation is strongly related to insulin resistance but not to impaired insulin secretion in a high risk population for diabetes. Metabolism.

[7] Pitsavos C, Tampourlou M, Panagiotakos DB, Skoumas Y, Chrysohoou C, Nomikos T, Stefanadis C (2007). Association between low-grade systemic inflammation and type 2 diabetes mellitus among men and women from the ATTICA Study. Rev Diabet Stud.

[8] Kolb H, Mandrup-Poulsen T (2005). An immune origin of type 2 diabetes?. Diabetologia.

[9] Demirtas L, Degirmenci H, Akbas EM, Ozcicek A, Timuroglu A, Gurel A, Ozcicek F (2015). Association of hematological indicies with diabetes, impaired glucose regulation and microvascular complications of diabetes. Int J Clin Exp Med.

[10] Budzianowski J, Pieszko K, Burchardt P, Rzeźniczak J, Hiczkiewicz J (2017). The role of hematological indices in patients with acute coronary syndrome. Dis Markers.

[11] Arikanoglu A, Yucel Y, Acar A, Cevik MU, Akil E, Varol S (2013). The relationship of the mean platelet volume and C-reactive protein levels with mortality in ischemic stroke patients. Eur Rev Med Pharmacol Sci.

[12] Hussain M, Babar MZ, Akhtar L, Hussain MS (2017). Neutrophil lymphocyte ratio (NLR): a well assessment tool of glycemic control in type 2 diabetic patients. Pak J Med Sci.

[13] Sefil F, Ulutas KT, Dokuyucu R (2014). Investigation of neutrophil lymphocyte ratio and blood glucose regulation in patients with type 2 diabetes mellitus. J Int Med Res.

[14] Kodiatte TA, Manikyam UK, Rao SB, Jagadish TM, Reddy M, Lingaiah HK, Lakshmaiah V (2012). Mean platelet volume in Type 2 diabetes mellitus. J Lab Physicians.

[15] KEY INDICATORS International Poverty Line(%) Non-Poor Poor Bottom 40 Top 60 [Internet]. http://chrome-extension://efaidnbmnnnibpcajpcglclefindmkaj/https://pdf.usaid.gov/pdf_docs/pdacg556.pdf.

[16] (2006). USAID. Nigeria economic performance assessment. https://pdf.usaid.gov/pdf_docs/PNADF350.pdf.

[17] American Diabetes Association (2013). Diagnosis and classification of diabetes mellitus. Diabetes Care.

[18] Alberti KGMM, Zimmet PZ (1998). Definition, diagnosis and classification of diabetes mellitus and its complications. Part 1: diagnosis and classification of diabetes mellitus provisional report of a WHO consultation. Diabet Med.

[19] Kirkwood R. B, Sterne A.C J. (2006). Study design, analysis and interpretation. Essential Medical Statistics, 2nd Edition.

[20] Mertoglu C, Gunay M (2017). Neutrophil-lymphocyte ratio and platelet-lymphocyte ratio as useful predictive markers of prediabetes and diabetes mellitus. Diabetes Metab Syndr.

[21] Zhang J, Shi X, Hao N (2018). Simvastatin reduces neutrophils infiltration into brain parenchyma after intracerebral hemorrhage via regulating peripheral neutrophils apoptosis. Front Neurosci.

[22] Mayyas FA, Al-Jarrah MI, Ibrahim KS, Alzoubi KH (2014). Level and significance of plasma myeloperoxidase and the neutrophil to lymphocyte ratio in patients with coronary artery disease. Exp Ther Med.

[23] Ridker PM, Cushman M, Stampfer MJ, Tracy RP, Hennekens CH (1997). Inflammation, aspirin, and the risk of cardiovascular disease in apparently healthy men. N Engl J Med.

[24] Isoda K, Young JL, Zirlik A (2006). Metformin inhibits proinflammatory responses and nuclear factor-kappaB in human vascular wall cells. Arterioscler Thromb Vasc Biol.

[25] Cameron AR, Morrison VL, Levin D (2016). Anti-inflammatory effects of metformin irrespective of diabetes status. Circ Res.

[26] Ekoru K, Doumatey A, Bentley AR (2019). Type 2 diabetes complications and comorbidity in sub-Saharan Africans. EClinicalMedicine.

[27] Kaushansky K (2005). The molecular mechanisms that control thrombopoiesis. J Clin Invest.

[28] Atak B, Aktas G, Duman TT (20191). Diabetes control could through platelet-to-lymphocyte ratio in hemograms. Rev Assoc Med Bras.

[29] Sharpe PC, Trinick T (1993). Mean platelet volume in diabetes mellitus. Q J Med.

[30] Ünübol M, Ayhan M, Güney E (2012). The relationship between mean platelet volume with microalbuminuria and glycemic control in patients with type II diabetes mellitus. Platelets.

[31] Hekimsoy Z, Payzin B, Örnek T (2004). Mean platelet volume in Type 2 diabetic patients. J Diabetes Complications.

[32] Lippi G, Salvagno GL, Nouvenne A, Meschi T, Borghi L, Targher G (2015). The mean platelet volume is significantly associated with higher glycated hemoglobin in a large population of unselected outpatients. Prim Care Diabetes.

[33] Ozder A, Eker HH (2014). Investigation of mean platelet volume in patients with type 2 diabetes mellitus and in subjects with impaired fasting glucose: a cost-effective tool in primary health care?. Int J Clin Exp Med.

[34] Korniluk A, Koper-Lenkiewicz OM, Kamińska J, Kemona H, Dymicka-Piekarska V (2019). Mean platelet volume (MPV): new perspectives for an old marker in the course and prognosis of inflammatory conditions. Mediators Inflamm.

[35] Schneider DJ (2009). Factors contributing to increased platelet reactivity in people with diabetes. Diabetes Care.

[36] Kakouros N, Rade JJ, Kourliouros A, Resar JR (2011). Platelet function in patients with diabetes mellitus: from a theoretical to a practical perspective. Int J Endocrinol.

[37] Biadgo B, Melku M, Abebe SM, Abebe M (2016). Hematological indices and their correlation with fasting blood glucose level and anthropometric measurements in type 2 diabetes mellitus patients in Gondar, Northwest Ethiopia. Diabetes Metab Syndr Obes.

[38] Sivri N, Tekin G, Yalta K, Aksoy Y, Senen K, Yetkin E (2013). Statins decrease mean platelet volume irrespective of cholesterol lowering effect. Kardiol Pol.

[39] Luzak B, Rywaniak J, Stanczyk L, Watala C (2012). Pravastatin and simvastatin improves acetylsalicylic acid-mediated in vitro blood platelet inhibition. Eur J Clin Invest.

[40] Dolasık I, Sener SY, Celebı K, Aydın ZM, Korkmaz U, Canturk Z (2013). The effect of metformin on mean platelet volume in dıabetıc patients. Platelets.

[41] Diaz-Morales N, Rovira-Llopis S, Bañuls C (2017). Does metformin protect diabetic patients from oxidative stress and leukocyte-endothelium interactions?. Antioxid Redox Signal.

